# Does Serum γKlotho Truly Provide Incremental Prognostic Value Beyond Established Risk Predictors in Multivessel Coronary Artery Disease?

**DOI:** 10.1002/clc.70410

**Published:** 2026-07-06

**Authors:** Mustafa Tunahan Öz, Umut Şahin, Adnan Kaya

**Affiliations:** ^1^ Department of Cardiology Bahçeşehir University Faculty of Medicine Istanbul Türkiye

Dear Editor,

We read with great interest the recently published study by Naman et al. evaluating serum γKlotho as a prognostic biomarker and incorporating it into a nomogram for predicting long‐term major adverse cardiovascular events (MACEs) in patients with multivessel coronary artery disease (MVD). The authors should be congratulated for assembling a relatively large cohort with both internal and external validation and for exploring an emerging biomarker that has received limited attention in cardiovascular medicine. Their work represents an important step toward identifying novel biological pathways associated with adverse outcomes in patients with complex coronary artery disease [1].

Nevertheless, we believe that one important issue deserves further discussion before serum γKlotho can be considered clinically useful as a prognostic biomarker.

Although γKlotho remained independently associated with MACEs after multivariable Cox regression and improved the discrimination of the proposed nomogram, the study did not determine whether γKlotho provides meaningful incremental prognostic information beyond conventional clinical variables already used in routine cardiovascular risk assessment. Demonstrating statistical significance within a multivariable model is not equivalent to demonstrating clinical usefulness [2, 3].

Current recommendations for biomarker evaluation emphasize that a novel marker should improve risk prediction beyond established clinical models. In addition to receiver operating characteristic analysis and C‐index assessment, contemporary studies are encouraged to evaluate metrics such as the Net Reclassification Improvement (NRI), Integrated Discrimination Improvement (IDI), and likelihood ratio testing. These analyses determine whether the addition of a new biomarker meaningfully reclassifies patients into more appropriate risk categories and provides clinically actionable information rather than merely statistical significance.

Another consideration relates to the biological interpretation of γKlotho. Unlike α‐Klotho, whose cardiovascular protective properties have been extensively investigated, γKlotho remains poorly characterized in cardiovascular disease. The present study interprets elevated circulating γKlotho as a marker of poor prognosis; however, it remains uncertain whether increased concentrations represent a causal pathogenic mechanism, a compensatory response to oxidative stress, or simply an epiphenomenon reflecting greater disease severity. Consequently, before incorporating γKlotho into clinical risk prediction models, mechanistic studies are required to clarify its biological role within ischemic cardiovascular disease [4].

Furthermore, the reported nomogram contains traditional predictors including age, diabetes mellitus, LDL cholesterol, left ventricular dimensions, and antiplatelet therapy, all of which already possess well‐established prognostic value. It would therefore be particularly informative to compare model performance before and after inclusion of γKlotho using formal model comparison techniques. Such analyses would allow readers to determine whether measurement of γKlotho would justify the additional laboratory cost and complexity in routine clinical practice.

Finally, external validation was performed in a second cohort from the same geographical region. Although this strengthens internal consistency, broader validation across different ethnic populations, healthcare systems, and contemporary treatment strategies will be essential before widespread implementation.

Overall, this well‐conducted study provides promising preliminary evidence supporting γKlotho as a potential biomarker in multivessel coronary artery disease. Future investigations incorporating incremental predictive metrics, prospective multicenter validation, and mechanistic studies will better establish whether γKlotho represents a clinically useful addition to existing cardiovascular risk stratification strategies rather than an interesting biological association.

We congratulate the authors for their valuable contribution and hope these comments stimulate further investigation into the clinical applicability of γKlotho in coronary artery disease.

## Data Availability

The data that support the findings of this study are available on request from the corresponding author. The data are not publicly available due to privacy or ethical restrictions.
